# Working memory loads differentially influence frame-induced bias and normative choice in risky decision making

**DOI:** 10.1371/journal.pone.0214571

**Published:** 2019-03-28

**Authors:** John M. Hinson, Paul Whitney, Cristina G. Wilson, Amy T. Nusbaum

**Affiliations:** 1 Department of Psychology, Washington State University, Pullman, WA, United States of America; 2 Department of Psychology, Temple University, Philadelphia, PA, United States of America; Temple University, UNITED STATES

## Abstract

Risky decision making can be biased by several types of contextual factors—in particular, framing of outcomes. A popular explanation for outcome framing effects is based on presumed affective reactions that contribute to accepting sure gains and avoiding sure losses. Other theories propose that selective weighting of information about gains and losses contributes to framing bias. Prior research on framing bias has focused on preferences rather than on decisions in which choices can be classified as advantageous (correct) or disadvantageous (incorrect) by a normative criterion. The current study used a novel hypothetical risky decision making task offering choices between a sure option and a gamble option. The gamble was advantageous or disadvantageous on different trials based on the normative criterion of expected value. Results showed risk avoidance with a gain frame and risk seeking with a loss frame, comparable to findings when choices involve preferences. We also examined the impact of working memory loads of either non-affective stimuli, most likely to interfere with acquisition of choice information, or affective stimuli, which might influence affective processes contributing to framing. The results were that non-affective working memory load produced the greatest framing magnitude, while affective load produced changes in framing magnitude across trials that varied by valence. In addition, only the non-affective load decreased advantageous choices and reduced the accuracy of answers to knowledge probe questions about the choices. The findings are consistent with the notion that framing effects may arise from cognitive non-engagement with the task, rather than arising by way of affective processes. Affective loads had a limited influence on framing and no reliable impact on choice accuracy or choice knowledge, suggesting that the affective loads influenced the weighting of choice information.

## Introduction

Human decision making is subject to many biases that represent violations of presumed normatively correct, or rational, decision making. One violation that is a popular object of study, framing bias, produces inconsistent decision making based on the way decision attributes, options, or outcomes of choices are presented [1]. Outcome framing in risky decision making, i.e., contrasting uncertain gains or losses with sure gains or losses, has been of particular interest since the pioneering work of Tversky and Kahneman [2]. Their early research used a prospect theory framework [3] to analyze factors responsible for violations of normatively correct decisions. According to the prospect theory interpretation, what is now routinely referred to as the framing effect, i.e., a bias toward risk seeking in a loss frame and a bias toward risk avoidance in a gain frame, is primarily a consequence of asymmetry in values of losses and gains, although probability weighting can also play a role [2].

While prospect theory and its modifications have received serious criticism [4], the theory remains an influential framework for examining factors responsible for framing effects. Recent extensions of the theory have examined the psychological basis for differences in weighting the value of gains and losses that could produce framing effects. Much of this work derives from the general dual process model that distinguishes between automatic, heuristic-based processes and controlled, deliberative processes [5, 6]. One illustration of the dual process account of framing effects is given by Kahneman and Frederick [7]. They argue that decision making is often dominated by an initial affective reaction, i.e., an affect heuristic [8, 9], which may be modified by deliberative cognitive control processes. According to this interpretation, framing effects result from initial automatic affective reactions associated with losses and gains, specifically, strong negative affect and the associated aversiveness of sure losses, and strong positive affect and associated attractiveness of sure gains. It follows that elimination of framing bias requires the suppression (or inhibition) of affective influences by cognitive control processes. Therefore, factors that interfere with cognitive control, but not affective processes, are expected to augment framing effects [10], while factors that reduce affect, but not cognitive control, are expected to diminish framing effects [8, 11]. Experimental examination of these factors, namely, manipulation of deliberative, non-affective control processes, or manipulation of automatic, affective processes, appear in a few studies.

Whitney, Rinehart, & Hinson [12] examined framing effects when cognitive control resources were influenced by means of a non-affective working memory (WM) load. This study used a dual task procedure, i.e., maintenance of a letter string, that has been applied in many settings [13, 14]. Their risky decision making task, adapted from De Martino, Kumaran, Seymour, & Dolan [15], involved a large number of decision trials, with some blocks of trials requiring the maintenance of a WM load, and other blocks requiring no WM load, while making decisions. Results of this study were that standard framing effects appeared in both load and no load trial blocks. Also, under WM load participants chose fewer gambles and had faster reaction time (RT). Whitney et al. interpreted these findings as inconsistent with Kahneman and Frederick’s [7] suggestion of greater influence of the affective heuristic system when the controlled, deliberative cognitive system is challenged. Instead, the results appeared consonant with work by Gonzalez, Dana, Koshino, & Just [16] indicating the role of a minimal effort, or satisficing, heuristic that interacts with both affective and controlled cognitive systems.

An experiment by Cassotti, Habib, Poirel, Aïte, Houdé, & Moutier [17], manipulated the context of affective reactions to choices in risky decision making. These researchers used a variant of the De Martino et al. [15] procedure along with presentations of pictures from the International Affective Picture System (IAPS) [18]. Different groups of participants were presented with affectively positive, affectively negative, or no IAPS pictures prior to making decisions in gain and loss frames. The standard framing effect occurred in both control and affectively negative groups, but no difference in gamble choices appeared between gain and loss frames in the affectively positive picture group. Relative to the control group, gamble choices for the affectively positive group declined in the loss frame but not in the gain frame. Cassotti et al. [17] concluded that priming by the affectively positive stimulus interfered with the automatic affective reaction normally occurring with a sure loss, thereby eliminating framing bias in that condition. In a related study, [19] examined the impact of incidental affective stimuli, in the form of pictures of angry or fearful faces, on framing bias in a risky decision making task. They reported incidental fear stimuli increased risk aversion in the gain frame, while incidental anger stimuli had the opposite effect.

Prior studies have established that non-affective or affective manipulations of the context of a risky decision can modify framing effects. What is currently unknown is whether these manipulations are modifying framing effects because they alter information *gating*, i.e., influencing throughput, retention, or availability of information required for a good decision, or because they alter information *weighting*, i.e., information is properly acquired and retained but is inappropriately weighted in comparison with other factors, such as affective processes. Either or both of information gating and information weighting could contribute to framing bias. Some results indicate that non-affective WM constraints can reduce the normalization of neural value representations in decision making [20], suggesting gating as a source. Others have argued that alterations in emotional processing, evident in affective disorders, can result in pronounced changes in the weighting of internal representations of utility [21, 22].

The current study was designed to extend prior findings on the role of affective and non-affective influences on risky decision making and frame-induced choice bias. We used a variant of a decision making task used by Morgan, Impallomeni, Pirona, and Rogers [23]. Similar to the task of Whitney et al. [12] and Cassotti et al. [17], it required repeated choices between gamble and sure options, presented in either a gain frame or loss frame. Unlike the prior studies, which focused on preference by primarily offering alternatives with equal expected value, the present study focused on choices in which either the sure option or the gamble option was normatively correct as defined by expected value of the gamble on each trial. That is, we examined risky choice *accuracy* rather than risky choice *preference*, by analyzing framing effects on both gambles and on correct choices, as defined by the normative criterion of expected value. At the end of each decision trial we asked questions about the choice options offered on that trial. These knowledge probes provided an assessment of the accuracy of choice information retained during the trial, shedding light on differences in framing attributable to gating or weighting of choice information.

Our first research objective was to determine if prior findings of framing effects with risky decision making choice preference could be replicated with a task based on normative choice accuracy. The second objective was to discover the degree to which affective and non-affective WM load facilitate or interfere with frame-induced choice bias. A third objective was to determine how well choices conformed to a normative criterion with or without different types of WM load. A fourth objective was to examine the relationship among choice knowledge, choice accuracy, framing bias, and WM load.

## Materials and methods

### Participants

Eighty adult volunteers from Washington State University and the local community in Pullman, WA, participated in the study. Participants were solicited by bulletin board postings and a Psychology Department website advertisement. This study was reviewed and approved for human subject participation by the Washington State University Institutional Review Board and included a written consent form that was signed by each person who agreed to participate in the study. Each participant was paid $15 for their service. The sample was 53% female, with age ranging from 18 to 28 years. Data from four research subjects were excluded from analysis due to failure to comply with task instructions.

### Procedure

Experimental tasks were programmed in EPrime (Psychology Software Tools) and run on individual desktop computer workstations. Morgan et al. [23] used a control gamble with a 50% probability and a consistent value of 10 points (50% chance of winning or losing 10 points), and an experimental gamble with varying probabilities (67% vs. 33%) and varying point values (+80, +20, -80, -20). The current task substituted a sure amount for the control gamble. Normatively correct choice types were 8 unique combinations of probability and amount for the gamble options from Morgan et al. [23]. These provided expected values of -47, -27.2, -13, -6.8, +6.8, +13, +27.2 and +47. Preference choice types were four unique combinations of amounts, each with a .5 probability. Choice options are provided in Table 1.

**Table 1:** Risky decision making choice options.

*Choices with normatively correct option*		
**Frame**	**Sure option**	**Gamble option**
Gain	+5	33% chance to gain $80 / 67% chance to lose $20
Gain	+5	33% chance to gain $80 / 67% chance to lose $80
Gain	+5	67% chance to gain $80 / 33% chance to lose $20
Gain	+5	67% chance to gain $80 / 33% chance to lose $80
Loss	-5	33% chance to gain $20 / 67% chance to lose $20
Loss	-5	33% chance to gain $20 / 67% chance to lose $80
Loss	-5	67% chance to gain $20 / 33% chance to lose $20
Loss	-5	67% chance to gain $20 / 33% chance to lose $80
*Choices with no normatively correct option*		
**Frame**	**Sure option**	**Gamble option**
Gain	+5	50% chance to gain $10 / 50% chance to lose $0
Gain	+40	50% chance to gain $80 / 50% chance to lose $0
Loss	-5	50% chance to lose $10 / 50% chance to lose $0
Loss	-40	50% chance to lose $80 / 50% chance to lose $0

Participants were given four practice trials to familiarize them with the procedure, and then commenced the decision making task with a hypothetical starting amount of $200. A wealth of decision making research supports the idea that participants respond to hypothetical and actual outcomes in a similar manner [24, 25, 26]. At the end of each trial a running tally was updated to represent the outcome of the choice made. If the sure gain or sure loss was chosen on the trial the tally was changed by the specified amount. If the gamble was chosen, the expected gain or loss from the gamble was used to update the tally. Participants completed 80 total choice trials. For each of four blocks of 20 trials, half of the trials were framed with sure gains and half with sure losses. Each block included eight trials where the gamble option was the correct choice and eight trials where the sure option was the correct choice, based on the normative criterion of expected value. The other four trials in each block were choices where the gamble and sure option had equal expected value. A deadline of 10 seconds was imposed for a choice response.

Participants were randomly assigned, with replacement to ensure equal group sizes, to one of four load groups with different demands for maintaining information in WM. Along with a no load control group, one group of participants was required to maintain a five item WM load with non-affective stimuli, i.e., a random letter set like that used by Whitney et al. [12], while other groups maintained a WM load of a single word that was either affectively positive or affectively negative. We presented words from the Affective Norms for English Words (ANEW) [27]. While IAPS pictures are commonly used as affective WM load, the effectiveness of ANEW words as affective stimuli is illustrated in a study using a variant of the Iowa Gambling Task (IGT) [28], where these stimuli were able to either facilitate or severely impair decision making depending on the congruency of the affective valence of the word with choice outcomes [29]. Moreover, ANEW words are less obtrusive than many IAPS pictures, which can be emotionally disturbing and capable of evoking strong, idiosyncratic memorial associations.

Affectively positive, affectively negative, and affectively neutral word sets were selected based on ratings in the ANEW system [27]. Positive and negative words fell between 1 and 2 *SD*s away from affectively neutral, while neutral words fell within 1 *SD* of affectively neutral. Words were equated for arousal and frequency of occurrence in the English language. A unique word appeared on each trial to avoid spurious contingencies arising from recognition of repeated words [29].

The four WM load groups are defined as follows.

*Control*—Participants completed each decision trial without being required to maintain additional information in WM.

*Non-affective*—Participants were required to maintain in WM five pseudorandom letters during each decision making trial. After making a decision, participants were asked to identify a letter in a randomly selected position among the set. Selection of the probe letter was pseudorandom with the constraint that across trials each position was probed equally often. Feedback on accuracy of the response to the probe was provided.

*Positive (Affective)*—Participants were required to maintain in WM a word with positive affective valence during each decision making trial. After the risky decision choice, participants were presented a word and asked if it was the same or different from the one they were maintaining. Same and different words were presented in pseudorandom order, with the constraint that each type appeared on half of the trials. Different words presented in the probe question were always affectively neutral. Feedback on accuracy of the response to the probe was provided.

*Negative (Affective)*—Participants had the same requirements as in the Positive load condition, except that a word with negative affective valence was presented to be maintained during each trial.

These groups were intended to permit the following comparisons on risky decision making in this task. The comparison of Control with the Non-affective load group would reveal the impact of taxing WM resources without directly manipulating affective processes with the load stimuli. The comparison of Control with either the affectively Positive or affectively Negative group would reveal the impact of a load that would not substantially tax cognitive resources, but that would manipulate affective reactions by way of the load stimuli. Thus, Non-affective load required the use of substantial WM resources, but did not directly influence the affective context of the decision. The two different affective loads, by design, placed minimal demands on WM resources, but manipulated the affective reactions orthogonal to the decision making process. The demonstrated importance of valence in prior decision making studies [17, 19] required affective loads differing in valence.

At the end of each trial participants were asked a knowledge probe question about the choice options offered on that trial. The knowledge probe required a true or false response to a question about either the sure option, e.g., “The sure option was $5”, or the gamble option, e.g., “The gamble option was 67% chance to win $20”. Questions about sure and gamble options appeared in pseudorandom order, with the constraint that half of the questions were about each. Feedback on accuracy of the response to the knowledge probe was provided.

In addition to the hypothetical risky decision making task all participants answered three questions about hypothetical scenarios to indicate their general risk preference, and completed a mood questionnaire based on 18 items of the Positive and Negative Affect Schedule (PANAS) [30].

**Fig 1:**
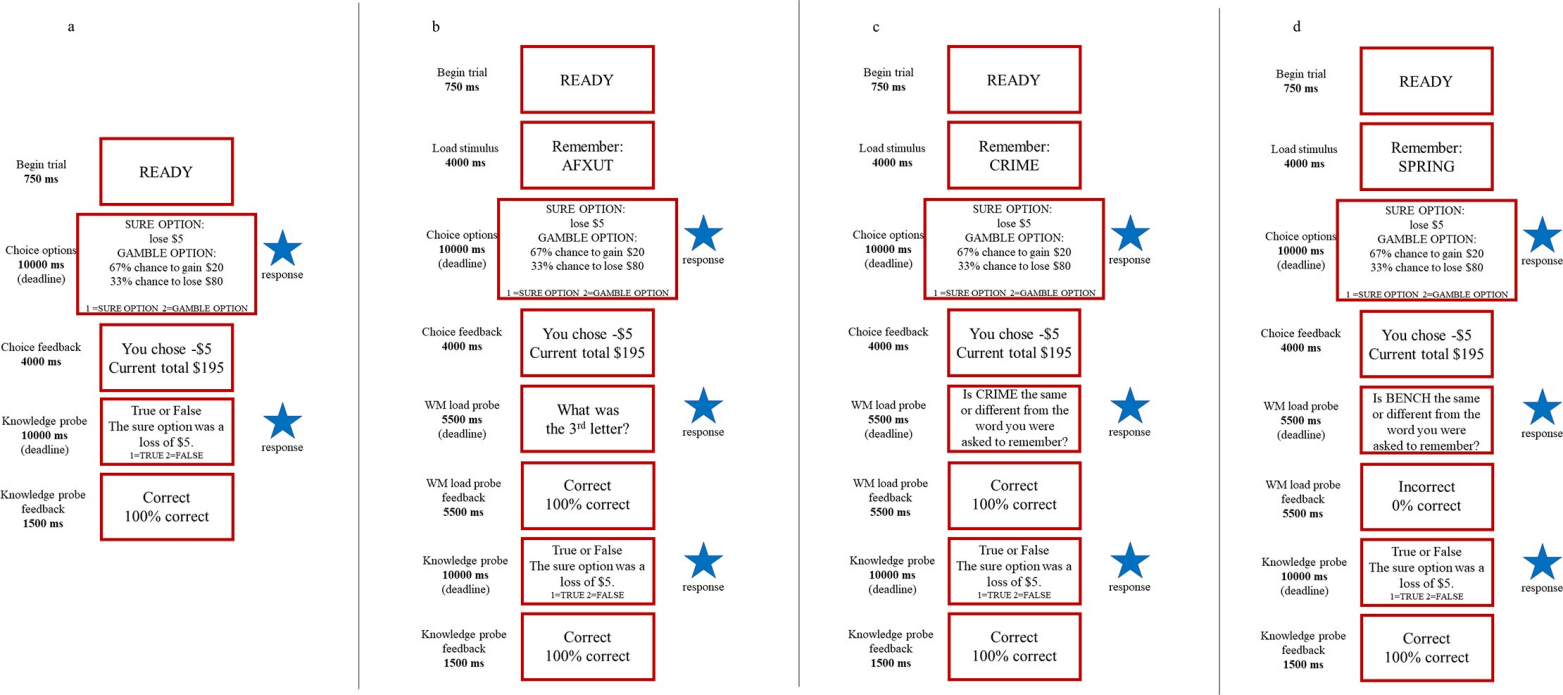
Schematic representation of trial events in the Control (1a), Non-affective (1b), Positive (1c) and Negative (1d) load condition. Participants pressed the ‘1’ key to choose the sure option or the ‘2’ key for the gamble.

In the schematic of the events during a decision making trial (Fig 1A–1D) note that for trial components requiring a response, i.e., decision making choice and probe question answers, there was a deadline. Responses prior to the deadline terminated the display and moved to the next component in the sequence. If no response was made within the deadline, something that occurred very rarely, feedback appeared indicating the deadline had passed and no response was recorded.

## Results

Participants in the Non-affective and Positive and Negative affective load conditions performed the task as intended, but at different levels of accuracy. Univariate Analysis of Variance (ANOVA), with alpha level set to .05, examining correct answers to load stimulus probe questions showed a significant effect of load, [*F*(2,54) = 27.048, *p* < .0001, *η*_*p*_^*2*^ = .500]. As expected, Non-affective load (M = .78, SD = .41) was more difficult to maintain than either the Positive load (M = .99, SD = .10) or Negative load (M = .98, SD = .16), confirmed with Tukey HSD post-hoc test for Non-affective versus Positive load (M_diff_ = .21, *p* = .001) and Non-affective versus Negative load (M_diff_ = .19, *p* = .001). Additional analysis revealed no significant effects of Frame or Type on accuracy of response to the load probe questions. In the results reported below, analysis is restricted to trials on which there is a normatively correct choice. The equal expected value trials were analyzed separately, but were too few in number to present meaningful results.

**Fig 2:**
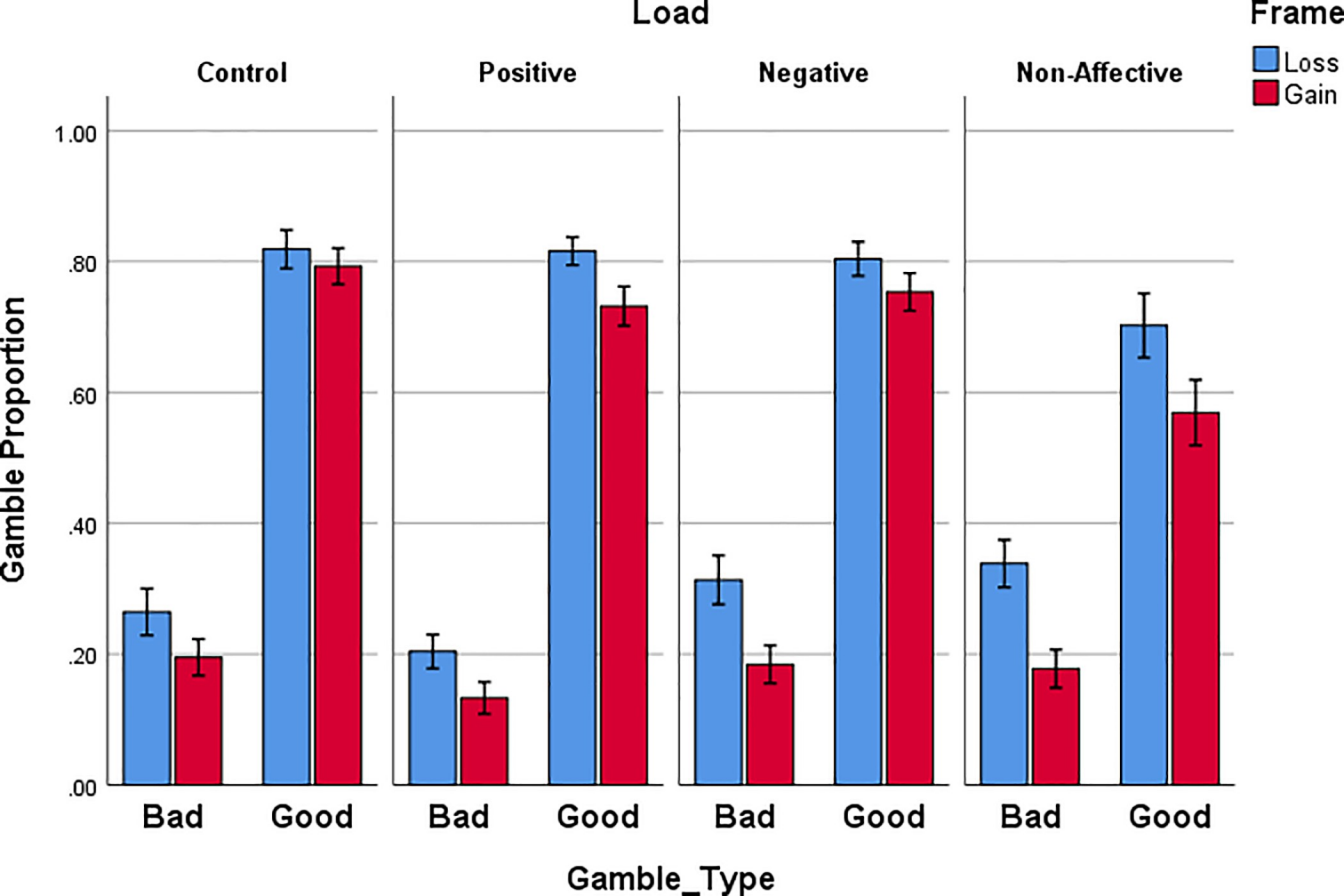
Proportion of good (normatively correct) and bad (normatively incorrect) gamble choices during each frame (Gain or Loss) for each WM load group. Errors bars represent +/- 1 standard error of the mean.

Gamble choices are designated as “good” when the gamble had a higher expected value than the sure option, or “bad” when the gamble had a lower expected value than the sure option. Participants frequently chose to gamble on good options and infrequently gambled on bad options (Fig 2). In addition, gambling was higher in the loss frame than in the gain frame. These results were confirmed with 2 (Frame) x 2 (Gamble Type [Good or Bad]) x 2 (Trial Block) x 4 (Load) repeated measures (RM) ANOVA. There were significant effects of Frame [*F*(1,72) = 39.603, *p* < .001, *η*_*p*_^*2*^ = .355] and Type [*F*(1,72) = 438.912, *p* < .0001, *η*_*p*_^*2*^ = .859]. Thus, standard framing effects were obtained for choices with a normatively correct option to gamble or not gamble. This standard framing bias did not prevent people from making good gamble choices on most trials.

The only reliable impact of WM load on overall gamble proportion was a Load x Type interaction, [*F*(3,72) = 4.123, *p* = .009, *η*_*p*_^*2*^ = .147]. Individuals in the Non-affective load group gambled less on good gamble trials. This was confirmed with a univariate ANOVA on good gamble choices [*F*(3,72) = 3.523, *p* = .019, *η*_*p*_^*2*^ = .128], with critical alpha corrected to .025 for two comparisons, along with a Tukey HSD for Control versus Non-affective load groups (M_diff_ = .17, *p* = .021).

**Fig 3:**
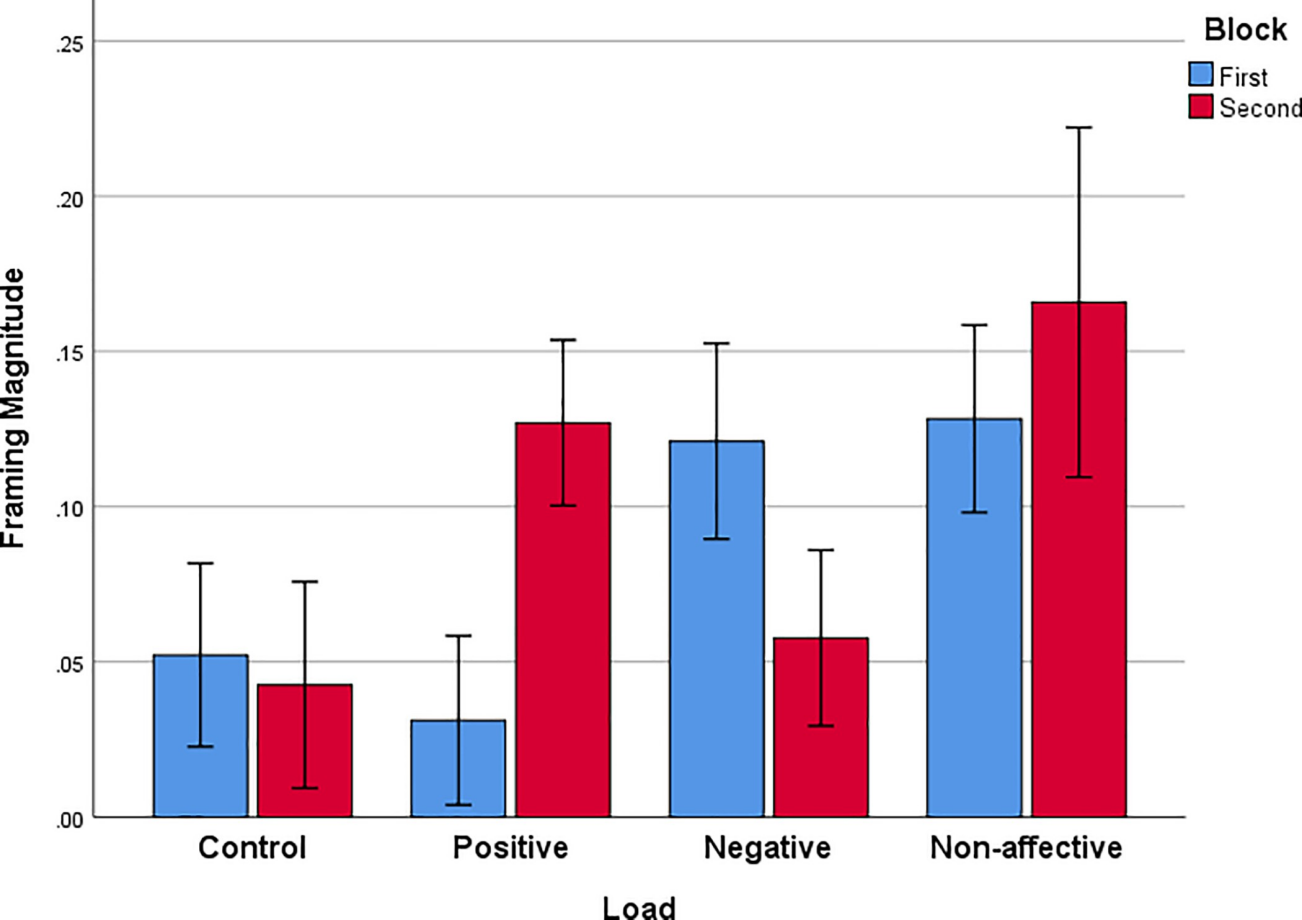
Magnitude of framing (gamble proportion in the gain frame minus gamble proportion in the loss frame) in two successive blocks of 40 trials for each WM load condition. Error bars represent +/- 1 standard error of the mean.

The magnitude of the framing effect was examined by taking the difference between proportion of gambles made during the gain frame and proportion of gambles made during the loss frame, across two successive blocks of 40 trials each (Fig 3). Simple contrasts between Control and each of the other load groups confirm that the Non-Affective load resulted in significantly larger framing magnitude than Control (Difference = .10, SEM = .04, *p* = .016). The magnitude of framing appeared to change over trial blocks for the Positive and Negative affective load groups. This change was evaluated by multiple, single sample t-tests, setting alpha to .00625 to correct for multiple comparisons. For the Positive group framing was absent in the first trial block but appeared in second trial block for Positive load, *t*(18) = 4.75, *p* < .001. For the Negative group framing was present in the first trial block, *t*(18) = 3.841, *p* < .001, but was not reliably present in the second block.

**Fig 4:**
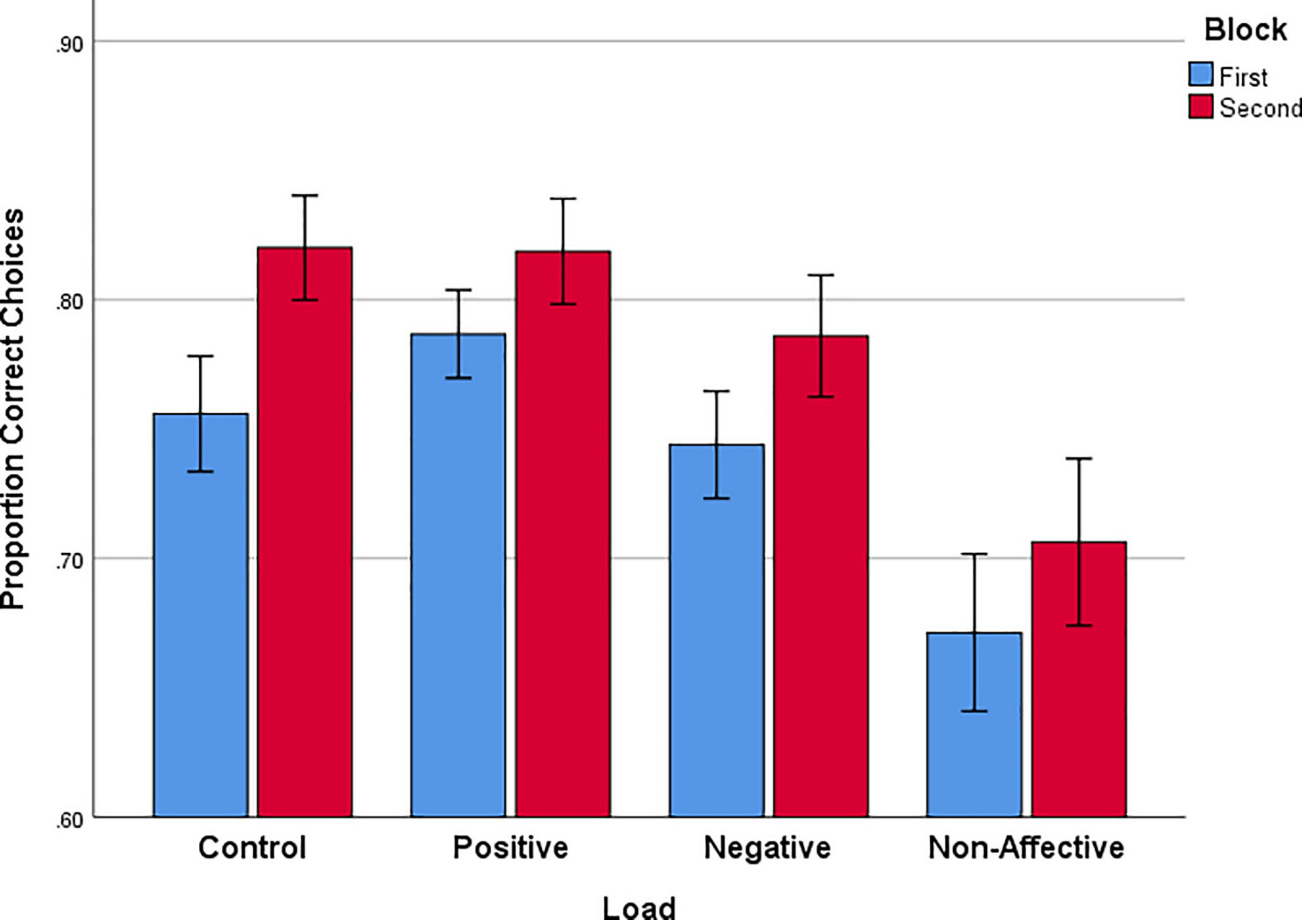
Proportion of correct choices (gamble when the gamble is good and taking the sure option when the gamble is bad) in two successive blocks of 40 trials for each WM load condition. Error bars represent +/- 1 standard error of the mean.

We now turn to how different WM loads may influence conformity to the normative choice criterion of expected value of the gamble. Proportion of normative correct choices, i.e., gambling when the gamble option is good and taking the sure option when the gamble is bad, across two successive trial blocks (Fig 4) shows that correct choices increased slightly across trial Block, *F*(1,72) = 18.168, *p* < .001, *η*_*p*_^*2*^ = .201. In addition, there is an interaction of gamble Type x Frame, *F*(1,72) = 40.627, *p* < .001, *η*_*p*_^*2*^ = .361. This interaction reflects the fact that more correct gambles are made in the loss frame, *t*(75) = 6.180, p < .001, and fewer correct gambles are made in the gain frame, *t*(75) = 3.873, *p* < .001, in all load conditions. There is also an effect of Load, *F*(3,72) = 3.985, *p* < .011, *η*_*p*_^*2*^ = .142. Tukey HSD multiple comparisons confirmed that correct choices in the Non-affective load condition were significantly lower than correct choices in either the Control (M_diff_ = .097, *p* = .036) or Positive load (M_diff_ = .118, *p* = .012) conditions.

A more refined way of evaluating the influences of load and framing bias on normative choice is to determine how well choices in each group conform to the expected value of gambles on each trial. A simple and direct means of assessing this conformity is obtained by fitting choices to a two-parameter binary logistic model, given by:

$$y=1/1+e^{‐(c+bx)}$$

where y is the binary probability of a gamble, x is the expected value of the gamble, e is the base of natural logarithms, and where parameter *c* is the intercept and *b* is the slope of the function. In this model gamble probability varies from 0 to 1 as a function of the eight unique expected values offered for the gamble option on different trials. Each of these unique expected values appeared equally often in the two successive blocks of trials, along with equally frequent presentation of the two frames. Table 2 provides the summary results from non-linear estimation of the binary logistic model, evaluating differences in WM load condition in each frame across two successive trial blocks. Estimates were obtained with the non-linear estimation model of SPSS (IBM SPSS Statistics, Version 24), using the sum of squared residuals for the loss function.

**Table 2:** Results from binary logistic regression model for gambles.

Load	Frame	Block	Parameter	Estimate	SEM	CI_lower	CI_upper
*Control*	*Loss*	*First*	*slope*	0.051	0.007	0.036	0.065
			*intercept*	0.404	0.137	0.136	0.673
		*Second*	*slope*	0.071	0.009	0.052	0.089
			*intercept*	0.174	0.131	-0.084	0.432
	*Gain*	*First*	*slope*	0.054	0.008	0.039	0.069
			*intercept*	0.081	0.130	-0.175	0.338
		*Second*	*slope*	0.074	0.010	0.054	0.094
			*intercept*	-0.128	0.135	-0.393	0.138
*Positive*	*Loss*	*First*	*slope*	0.064	0.009	0.046	0.081
			*intercept*	0.137	0.133	-0.124	0.398
		*Second*	*slope*	0.076	0.010	0.056	0.096
			*intercept*	0.030	0.133	-0.232	0.291
	*Gain*	*First*	*slope*	0.056	0.008	0.040	0.071
			*intercept*	-0.127	0.130	-0.384	0.129
		*Second*	*slope*	0.064	0.009	0.047	0.081
			*intercept*	-0.740	0.148	-1.030	-0.450
*Negative*	Loss	*First*	*slope*	0.042	0.006	0.030	0.055
			*intercept*	0.398	0.131	0.139	0.656
		*Second*	*slope*	0.056	0.008	0.040	0.072
			*intercept*	0.226	0.133	-0.036	0.487
	*Gain*	*First*	*slope*	0.057	0.008	0.041	0.072
			*intercept*	-0.212	0.132	-0.471	0.048
		*Second*	*slope*	0.061	0.008	0.044	0.078
			*intercept*	-0.165	0.132	-0.425	0.096
*Non-affective*	*Loss*	*First*	*slope*	0.028	0.005	0.018	0.038
			*intercept*	0.103	0.122	-0.138	0.343
		*Second*	*slope*	0.033	0.006	0.022	0.044
			*intercept*	0.079	0.124	-0.165	0.323
	*Gain*	*First*	*slope*	0.032	0.006	0.021	0.043
			*intercept*	-0.509	0.132	-0.767	-0.250
		*Second*	*slope*	0.039	0.006	0.027	0.051
			*intercept*	-0.715	0.141	-0.992	-0.437

**Fig 5:**
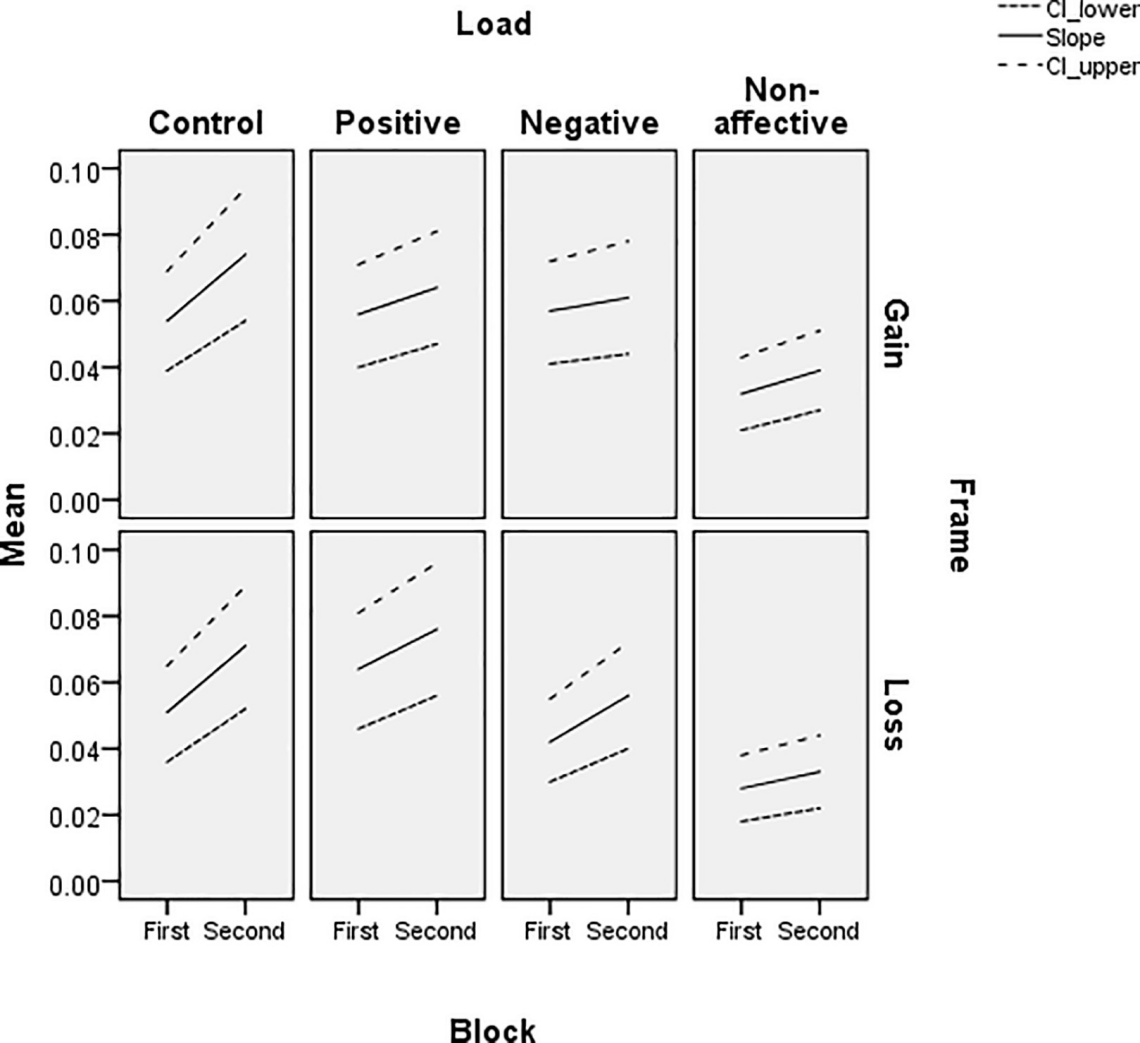
Estimated slope and upper and lower bounds for 95% confidence interval for binary logistic regression of gambles, in each frame and trial block, for each WM load condition.

**Fig 6:**
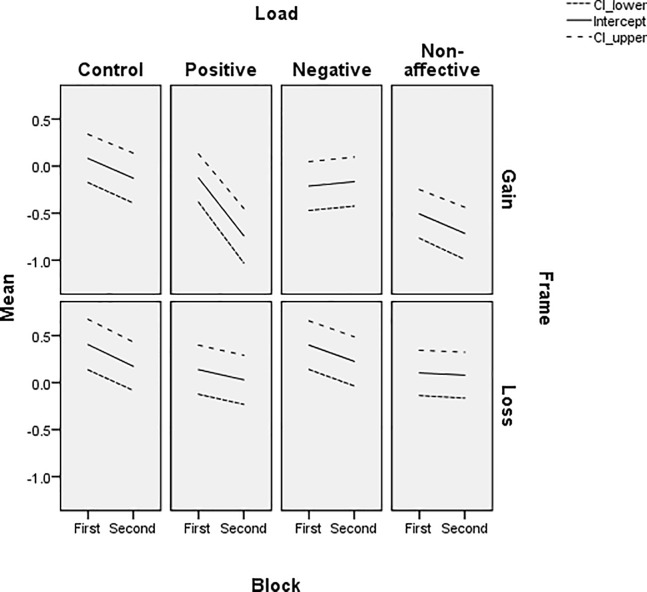
Estimated intercept and upper and lower bounds for 95% confidence interval for binary logistic regression of gambles, in each frame and trial block, for each WM load condition.

The slope (Fig 5), reflecting sensitivity to the normative choice criterion, is stable for all WM load conditions and there is a trend for increases from the first to second trial block. The Non-affective load group has the lowest sensitivity to the normative criterion, although the separation of this condition from the Positive and Negative affective load groups is less distinct in the first trial block and the gain frame. Thus, sensitivity to the normative criterion is reduced by Non-affective WM load, but not by the Positive or Negative affective load. There was no apparent difference in sensitivity between gain and loss frames, despite the large effect of frame on gambles. The influence of frame appears in the intercept (Fig 6), with relatively more positive values in the loss frame and relatively more negative values in the gain frame for all WM load groups. While reasonably stable, the parameter does show the reliable change in framing bias from early to later trials in the Positive load group. Thus, Non-affective WM load is the only condition that affects sensitivity to the normative choice criterion, despite the fact that there are marked differences in frame-induced bias in gambles across the four WM load groups.

**Fig 7:**
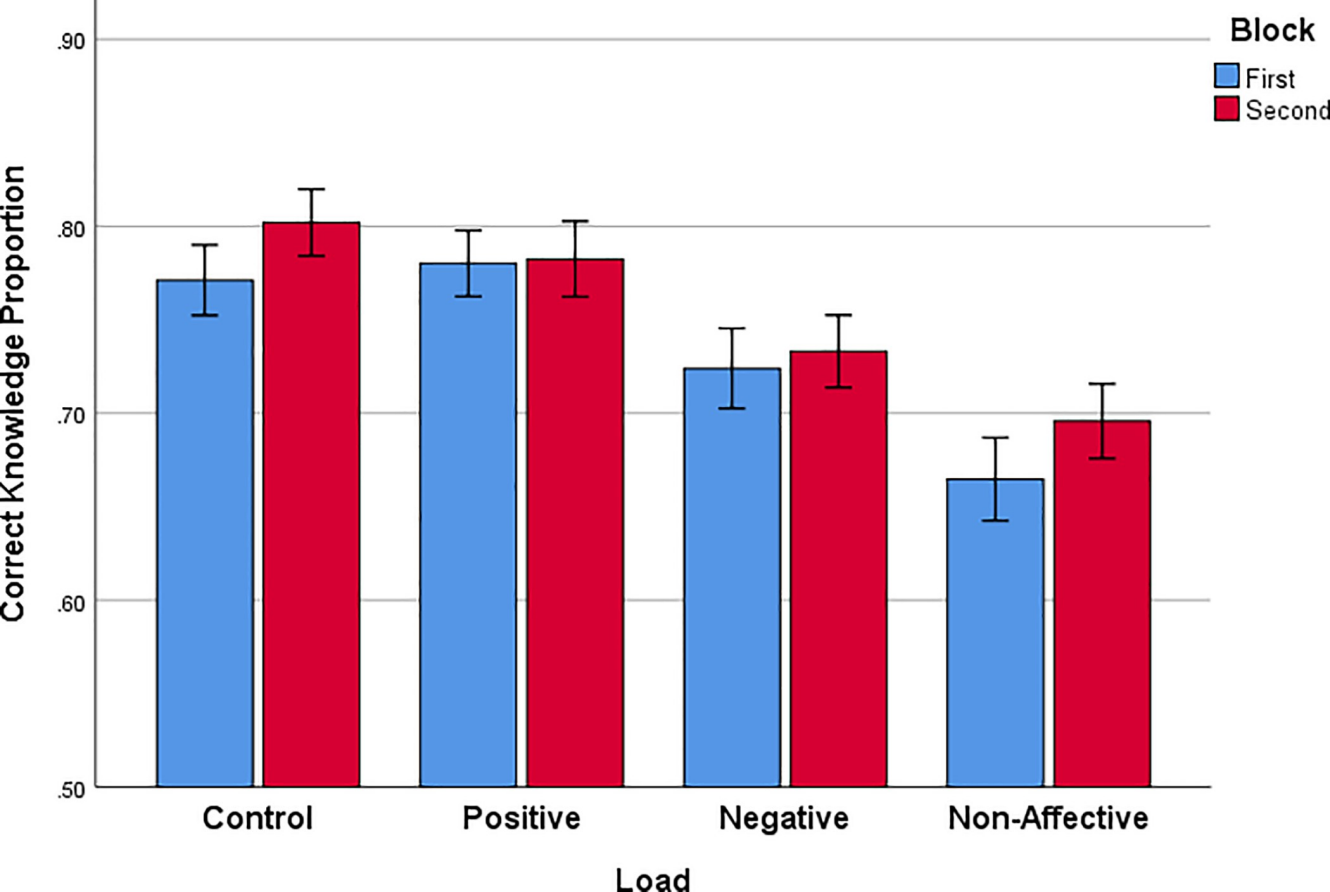
Proportion of correct responses to knowledge probe questions in two successive blocks of trials for each WM load condition. Error bars represent +/- 1 standard error of the mean.

Turning to knowledge of choices (Fig 7), accuracy of answers to probe questions increased slightly across trial blocks, evaluated by RM ANOVA of probe question accuracy, [*F*(1,72) = 13.704, *p* < .0001, *η*_*p*_^*2*^ = .160]. In addition, there was an effect of WM load, [*F*(3,72) = 5.114, *p* < .003, *η*_*p*_^*2*^ = .176]. Tukey HSD multiple comparisons indicate knowledge accuracy for Control (M_diff_ = .1053, p = .005) and Positive load (M_diff_ = .0987, *p* = .01) was reliably greater than the Non-affective load condition. Thus, the group with the largest and most consistent framing effects, and with the lowest sensitivity to the normative choice criterion of expected value, also had the worst knowledge accuracy.

**Fig 8:**
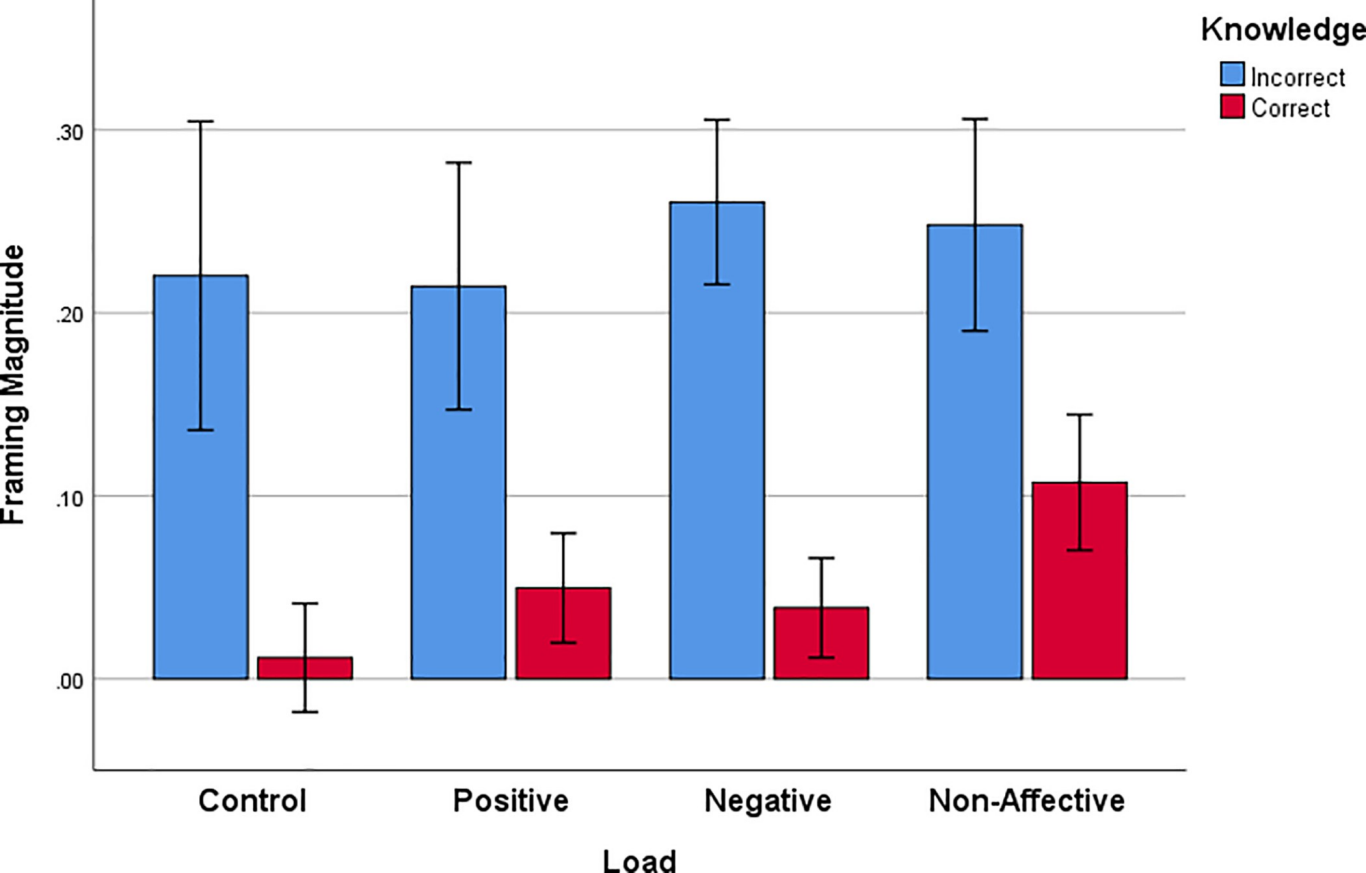
Magnitude of framing (gamble proportion in the gain frame minus gamble proportion in the loss frame) for trials with either incorrect or correct answers to knowledge probe questions. Error bars represent +/- 1 standard error of the mean.

Knowledge accuracy is also associated with magnitude of framing. This can be shown by comparing framing magnitude on trials where knowledge probe answers were accurate or inaccurate (Fig 8). Framing magnitude is large for all groups on trials where knowledge probe answers are incorrect and small on trials with correct knowledge answers, resulting in a significant effect of knowledge probe Accuracy, [*F*(1,72) = 27.312, *p* < .001, *η*_*p*_^*2*^ = .275].

**Fig 9:**
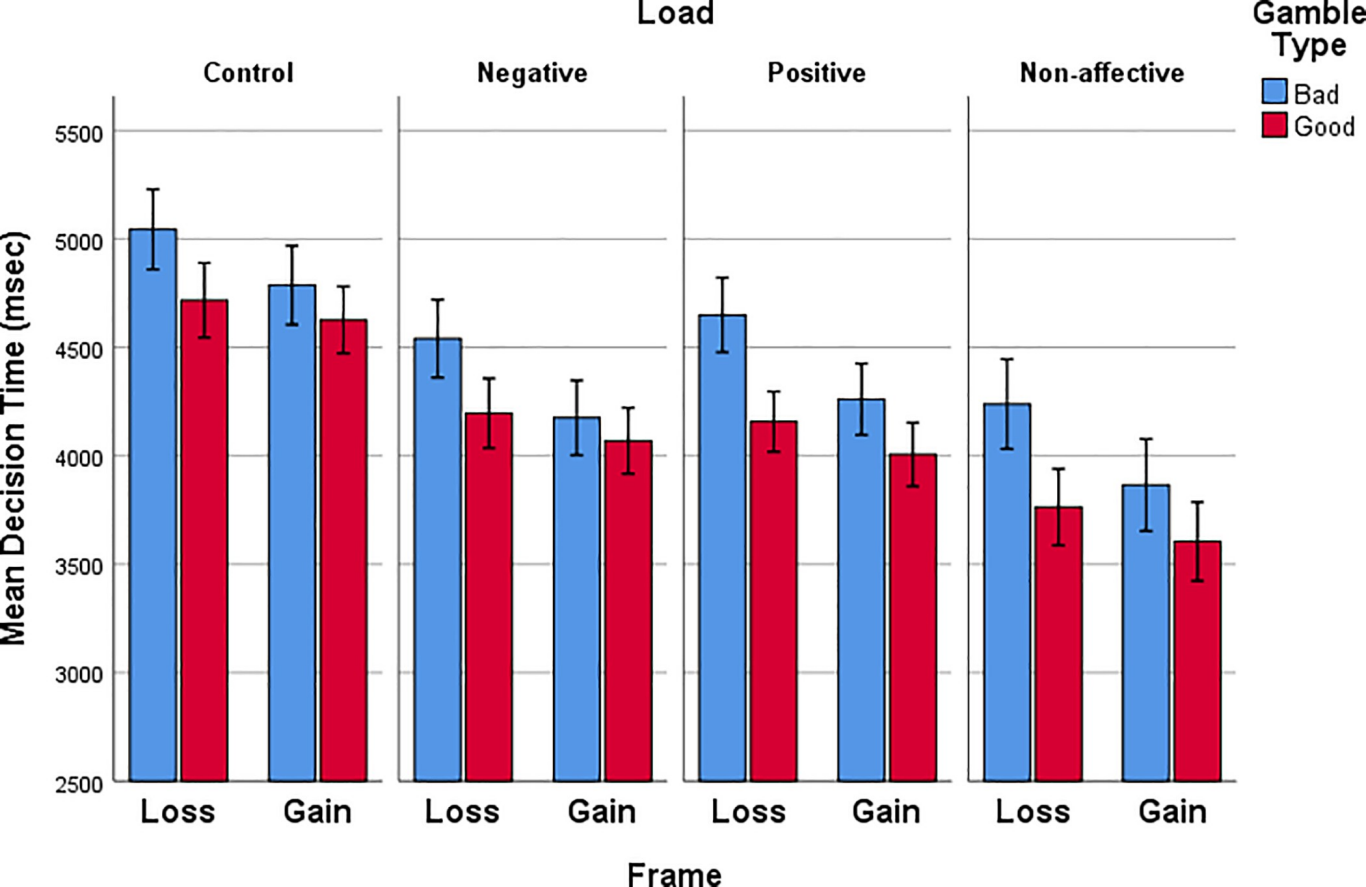
Decision time (msec) during the gain and loss frames for each gamble type (good or bad gamble) for all WM load groups. Error bars represent +/- 1 standard error of the mean.

Finally, we examine how decision time may be related to the WM load group differences in the performance described above. Decision time (Fig 9) for good and bad gamble choices in different frames shows a distinctive pattern for all WM load groups. Participants took longer to make decisions in the loss frame compared to the gain frame. In addition, they took longer to make a decision when the gamble option was bad compared to when the gamble was good. This appears as a significant interaction of Frame X gamble Type, [*F*(1,72) = 12.923, *p* < .001, *η*_*p*_^*2*^ = .152]. There was also an effect of Load, [*F*(1,72) = 3.371, *p* < .023, *η*_*p*_^*2*^ = .123], in which decision time during Non-affective load was faster than Control, Tukey HSD (M_diff_ = 925, *p* = .012).

## Discussion

In terms of our original research objectives, we found that risky decision choices with normatively correct and incorrect options resulted in standard framing effects. These results are consistent with the prior findings [12] examining choice preference. Compared with a no load Control group, a Non-affective WM produced large, consistent framing effects. Affective WM loads, Positive or Negative, did not systematically enhance framing, although affective valence did have some impact that was generally consistent with prior reports [17, 19, 31, 32].

Apart from the presence of the framing effect, individuals in this study generally chose good gambles and made choices that were sensitive to the normative criterion of expected value. Choice accuracy was only impaired by the Non-affective load, a finding consistent with the deleterious impact of non-affective WM loads in experiential decision making tasks [14, 29]. In addition, participants provided reasonably accurate answers to questions about choice probability and amount information on each trial. Non-affective WM load was the only condition that impaired overall knowledge accuracy.

Framing is generally presumed to be based on affective processes, but we found no evidence that affective load stimuli augmented framing bias and no evidence that affective valence of load stimuli asymmetrically influenced framing bias. Recent research by Li et al. [33] provides an interpretation of framing that helps to explain the current findings. While Li et al. agree that affective processes may contribute to framing in decision making, they argue that framing effects are reflective of different levels of cognitive engagement and that the appearance of framing does not require an interaction between cognitive engagement and affective processes. One source of evidence in their study was response time data. Specifically, they found that choices made during a loss frame took longer that choices made during a gain frame. Moreover, choices that were inconsistent with the frame, i.e., gambling during the gain frame or not gambling the loss frame, took longer than choices consistent with frame. While increased response time is not always indicative of increased cognitive effort [34], it can be in some cases. Li et al. provided neural activation data to bolster their claim that level of cognitive effort was a primary causal factor for framing in their study.

We found a comparable pattern of decision times in our study, in that decision time in the loss frame was longer than in the gain frame, decision time for bad gamble options was longer than for good gamble options, and decision time for bad gamble options in the loss frame was longest of all. The group in which framing was most prominent was the Non-affective load group, and it was this group that had much shorter decision times. A reasonable interpretation, consistent with Li et al., is that the Non-affective WM load reduced cognitive engagement, allowing shorter decision times in the primary decision task to accommodate the demands of maintaining the digit load in the secondary task. The reduced cognitive engagement contributed to more frequent reversion to a default mode of processing, relying on the pre-existing framing bias to determine choices.

This interpretation is also concordant with two aspects of our knowledge probe results. First, along with shortest decision times, overall knowledge accuracy was lowest in the Non-affective WM load group. This suggests that knowledge was not acquired, or not retained, during more trials in the Non-Affective load group because of demands on WM resources that limited the level of cognitive engagement. Second, across all load groups we found that framing magnitude was large on trials where knowledge was inaccurate and small on trials with accurate knowledge. This suggests that cognitive engagement, trial by trial, was a primary contributor to the magnitude of framing in all WM load groups, but most prominently in the group for whom WM resources were most compromised. Thus, the increased magnitude of framing, the decreased choice accuracy, and the decreased knowledge accuracy in the current study can all explained by adaptations of cognitive processing that minimize the impact of increased cognitive load.

Although the affective WM loads used in this study did not affect choice accuracy, they did influence framing magnitude in distinct ways. These findings add to a variety of reports that affective loading may be a significant factor in decision making without evocation of a specific emotional state or mood [35, 36, 37]. However, the distinctive time course of framing magnitude for affective loads in our study remains to be explained. Like prior reports [17] we found that a positive affective load initially eliminated framing, but framing appeared in the later block of trials. The opposite pattern was observed with Negative affective load, in that framing magnitude was large initially and diminished later on. The simplest explanation for a time-based change in framing would be that participants habituated to repeated presentations of the affective words [38]. However, if habituation were a part of the cognitive control mechanisms modifying the impact of affective factors on decision making as postulated by others [7], the magnitude of framing would be expected to decline rather than increase over time. Thus, habituation to affective stimuli is consistent with results for negative affective load, but inconsistent with the results for positive affective load.

Remaining questions about the influence of the affective loads could be addressed in future research that removes some of the limitations of the current study. First, because of our relatively small sample sizes for WM load groups, it will be important to eliminate the possibility that what we observed were simply idiosyncratic reactions to the affective words that would not generalize to a larger sample. Second, further research should directly measure affective reactions to positive and negative stimuli used in the Affective loads. Although we had good reason to believe that affective reactions were being produced by words we employed, based on the original work that established the ANEW set [27], based on the use of this word set in other decision making studies [29], and based on pilot studies preceding the current experiment, it would still strengthen the findings to have a direct physiological measure of affect in this decision making setting. Future studies should also try to extend our work by using tasks where actual stakes of money are involved, as opposed to the hypothetical stakes that were used in this study. While previous work shows a high correlation between real and hypothetical gambles, this should still be assessed in this specific context. Finally, in the current study we separately examined a cognitively demanding Non-affective WM load and a cognitively undemanding pair of Affective WM loads. To verify the critical role of cognitive engagement, it would be necessary to design load manipulations so that the potential interactive and additive effects of cognitive engagement and affective reactions can be unambiguously analyzed.
